# Early Postnatal Metabolic Profile in Neonates With Different Birth Weight Status: A Pilot Study

**DOI:** 10.3389/fped.2021.646860

**Published:** 2021-04-27

**Authors:** Serdar Beken, Saygin Abali, Neslihan Yildirim Saral, Bengisu Guner, Taha Dinc, Eda Albayrak, Melike Ersoy, Meltem Kilercik, Muge Halici, Ezgi Bulbul, Didem Kaya, Melis Karabay, Zeynep Alize Ay, Gulten Zeynep Eksi, Fehime Benli Aksungar, Ayse Korkmaz, Mustafa Serteser

**Affiliations:** ^1^Department of Pediatrics, Section of Neonatology, School of Medicine, Acibadem Mehmet Ali Aydinlar University, Istanbul, Turkey; ^2^Department of Pediatrics, Section of Pediatric Endocrinology, School of Medicine, Acibadem Mehmet Ali Aydinlar University, Istanbul, Turkey; ^3^Department of Metabolism, Acibadem Labmed Clinical Laboratories, Istanbul, Turkey; ^4^Department of Pediatrics, School of Medicine, Acibadem Mehmet Ali Aydinlar University, Istanbul, Turkey; ^5^School of Medicine, Acibadem Mehmet Ali Aydinlar University, Istanbul, Turkey; ^6^Department of Pediatrics, Bakirkoy Dr Sadi Konuk Training and Research Hospital, University of Health Sciences, Istanbul, Turkey; ^7^Department of Biochemistry, School of Medicine, Acibadem Mehmet Ali Aydinlar University, Istanbul, Turkey

**Keywords:** small for gestation age, large for gestational age, carnitine, metabolomics (OMICS), tandem MS

## Abstract

**Introduction:** Restricted or enhanced intrauterine growth is associated with elevated risks of early and late metabolic problems in humans. Metabolomics based on amino acid and carnitine/acylcarnitine profile may have a role in fetal and early postnatal energy metabolism. In this study, the relationship between intrauterine growth status and early metabolomics profile was evaluated.

**Materials and Methods:** A single-center retrospective cohort study was conducted. Three hundred and sixty-one newborn infants were enrolled into the study, and they were grouped according to their birth weight percentile as small for gestational age (SGA, *n* = 69), appropriate for gestational age (AGA, *n* = 168), and large for gestational age (LGA, *n* = 124) infants. In all infants, amino acid and carnitine/acylcarnitine profiles with liquid chromatography-tandem mass spectrometry (LC-MS/MS) were recorded and compared between groups.

**Results:** LGA infants had higher levels of glutamic acid and lower levels of ornithine, alanine, and glycine (*p* < 0.05) when compared with AGA infants. SGA infants had higher levels of alanine and glycine levels when compared with AGA and LGA infants. Total carnitine, C0, C2, C4, C5, C10:1, C18:1, C18:2, C14-OH, and C18:2-OH levels were significantly higher and C3 and C6-DC levels were lower in SGA infants (*p* < 0.05). LGA infants had higher C3 and C5:1 levels and lower C18:2 and C16:1-OH levels (*p* < 0.05). There were positive correlations between free carnitine and phenylalanine, arginine, methionine, alanine, and glycine levels (*p* < 0.05). Also, a positive correlation between ponderal index and C3, C5-DC, C14, and C14:1 and a negative correlation between ponderal index and ornithine, alanine, glycine, C16:1-OH, and C18:2 were shown.

**Conclusion:** We demonstrated differences in metabolomics possibly reflecting the energy metabolism in newborn infants with intrauterine growth problems in the early postnatal period. These differences might be the footprints of metabolic disturbances in future adulthood.

## Introduction

Restricted or enhanced intrauterine growth is associated with elevated risks of early and late metabolic problems (1–3). Inadequate glycogen, reduced sources for gluconeogenesis and, in some cases, raised insulin levels are responsible for hypoglycemia and energy deprivation in newborn infants with fetal growth restriction and, postnatally, who were defined as small for gestational age (SGA) infants (4). Also, newborns with enhanced intrauterine growth, namely large for gestational age (LGA) infants, have increased risk of hypoglycemia and energy failure in the postnatal life (5).

Studies focusing on the relationship between metabolomics and fetal growth have emerged quickly in the past years. It has been thought that early postnatal metabolic profiles of low birth weight (LBW) and macrosomic babies might differ from the normal (6, 7). Metabolomics based on amino acid and carnitine/acylcarnitine profile studies with liquid chromatography-tandem mass spectrometry (LC-MS/MS) may have a role in our understanding of fetal and early postnatal energy metabolism.

Carnitine (3-hydroxy-4-trimethylazaniumyl butanoate) is a water-soluble amino acid derivative that plays an important role in energy metabolism. The major functions of carnitine are the transport of activated long-chain fatty acids (LCFA) from the cytosol to the mitochondrial matrix for β-oxidation, transfer of products of peroxisomal β-oxidation to the mitochondria for oxidation in the citrate cycle, modulation of the acylcoenzyme A (CoA)/CoA ratio, and storage of energy as acylcarnitine (8, 9).

During prolonged fasting, glycogen stores are depleted, and alternative glucose supply is necessary to maintain normal blood glucose level. A major alternative metabolic pathway is gluconeogenesis which is described as the generation of glucose from certain non-carbohydrate carbon substrates such as glycerol, lactate, pyruvate, and glucogenic amino acids (10). Also, mitochondrial fatty acid β-oxidation is the major energy source in this condition. In addition to its role in detecting many inborn metabolic errors, amino acid, and carnitine/acylcarnitine profile with LC-MS/MS (11) may be used to identify the associations between these energy pathways in SGA and LGA newborns.

The aim of this study was to evaluate the relationship between early postnatal amino acid and carnitine/acylcarnitine profiles and intrauterine growth status in newborn infants.

## Materials and Methods

A single-center retrospective pilot study was conducted between January 2016 and December 2019 in Acibadem Mehmet ali Aydinlar University, Atakent Hospital, Istanbul, Turkey. During the study period, only inborn infants who were fed exclusively with breast milk in the first 24–48 h of life were included in the study. Infants born to mothers with gestational diabetes, preeclempsia, infections, and other diseases; formula-fed infants; multiple gestations; preterm infants (gestational age below 37^0/7^ weeks); and newborn infants who had any congenital anomaly were excluded from the study to avoid their confounding effects. Patients with inborn metabolic diseases detected by expanded newborn screening program (ENSP) were also excluded. Birth weight (BW) standard deviation scores (SDS)/percentiles of all newborn infants were calculated according to national references by the Child Metrics (12, 13). Infants were grouped according to their BW SDS/percentile. The groups were as follows: *group 1*, infants BW below the 10th percentile (SGA); *group 2*, BW between the 25th and 75th percentiles appropriate for gestational age (AGA); and *group 3*, infants BW above the 90th percentile (LGA). Group 1 was also divided into two subgroups as SGA below < -2 SDS and SGA between −2 SDS and 10th percentile.

Demographic and clinical data from hospital electronic medical records including maternal smoking status, gestational age, gender, BW, crown–heel length at birth (BL), and occipitofrontal head circumference (HC) were recorded.

Dried blood spots (DBSs) for ENSP were taken between postnatal 24th and 48th h after at least 24–28 h of exclusive breastfeeding in every 3 h.

The results of amino acids, including alanine [reference value (RV): 90–900 μmol/L], methionine (RV: 9.0–65 μmol/L), phenylalanine (RV: 22.9–120 μmol/L), tyrosine (RV: 26.9–275 μmol/L), leucine/isoleucine (RV: 43.4–373 μmol/L), valine (RV: 52.8–250 μmol/L), arginine (RV: 0.0–50 μmol/L), citrulline (RV: 2.0–65 μmol/L), glycine (RV: 105–1,100 μmol/L), ornithine (RV: 19.7–300 μmol/L), arginino-succinic acid (RV: 0.0–0.66 μmol/L), glutamic acid (RV: 0.0–704 μmol/L), and also carnitine/acylcarnitine profile including free carnitine (C0) (RV: 8.6–90.0 μmol/L), acetyl carnitine (C2) (RV: 5.0–73.4 μmol/L), propionyl carnitine (C3) (RV: 0.0–6.8 μmol/L), butyryl carnitine (C4) (RV: 0.0–1.2 μmol/L), isovaleryl carnitine (C5) (RV: 0.0–0.6 μmol/L), tiglyl carnitine (C5:1) (RV: 0.0–0.13 μmol/L), hexonyl carnitine (C6) (RV: 0.0–0.21 μmol/L), octanoyl carnitine (C8) (RV: 0.0–0.32 μmol/L), decanoyl carnitine (C10) (RV: 0.0–0.48 μmol/L), decenoyl carnitine (C10:1) (RV: 0.0–0.28 μmol/L), dodecanoyl carnitine (C12) (RV: 0.0–0.69 μmol/L), tetradecanoyl carnitine (C14) (RV: 0.0–0.8 μmol/L), tetradecenoyl carnitine (C14:1) (RV: 0.0–0.6 μmol/L), tetradecadienoyl carnitine (C14:2) (RV: 0.0–0.25 μmol/L), palmitoyl carnitine (C16) (RV: 0.0–8.70 μmol/L), palmitoleyl carnitine (C16:1) (RV: 0.0–1.04 μmol/L), stearyl carnitine (C18) (RV: 0.0–2.24 μmol/L), oleyl carnitine (C18:1) (RV: 0.0–2.8 μmol/L), linolenoyl carnitine (C18:2) (RV: 0.0–0.9 μmol/L), glutaryl carnitine (C5-DC) (RV: 0.0–0.21 μmol/L), methylglutaryl carnitine (C6-DC) (RV: 0.0–0.20 μmol/L), 3-OH butyryl carnitine (C4-OH) (RV: 0.0–0.48 μmol/L), 3-OH isovaleryl carnitine (C5-OH) (RV: 0.0–0.80 μmol/L), 3-OH tetradecanoyl carnitine (C14-OH) (RV: 0–0.12 μmol/L), 3-OH palmitoyl carnitine (C16-OH) (RV: 0.0–0.1 μmol/L), 3-OH palmitoleyl carnitine (C16:1-OH) (RV: 0.0–0.18 μmol/L), 3-OH oleyl carnitine (C18:1-OH) (RV: 0.0–0.1 μmol/L), and 3-OH linonenoyl carnitine (C18:2-OH) (RV: 0.0–0.1 μmol/L) with LC-MS/MS were compared in each group. The sum of all carnitine and acylcarnitines was presented as total carnitine.

### Sample Preparation and LC-MS/MS Analysis

The method is given in a previous study (14); briefly, DBSs were punched as 3.2-mm diameter disc from filter paper [Schleicher and Schuell (S&S) no 903] and put into a 96-well-polypropylene microwell plate. A standard solution containing methanol and a mixture of isotope-labeled internal standard (IS) for amino acids and acylcarnitines (200 μl in total) was added to each well. Cambridge Isotopes (USA) IS set A (Cat. No: NSK-A-1) for amino acids and set B (Cat. No: NSK-B-1) for acylcarnitines were used in the analysis (14). Sample extraction was performed by a rotary shaker for 30 min at room temperature. The extracted samples were transferred to another plate. Sixty microliters of butanolic HCl was added into each well and incubated for 25 min at 65°C for derivatization. After the samples were evaporated at 45°C for 1 h, 100 μl of mobile phase (acetonitrile:water 80:20 v/v) was added and shaken for 10 min, and finally, the analysis was carried out. Dried blood spot quality control materials for amino acids and acylcarnitines were obtained from the Centers for Disease Control and Prevention [CDC, Atlanta, GA, USA, Newborn Screening Quality Assurance Program (NSQAP)].

Analysis of amino acids and acylcarnitines in the DBS sample by flow injection analysis (FIA) was performed using the Shimadzu LCMS-8040 Liquid Chromatograph Triple Quadrupole Mass Spectrometer (Shimadzu Corporation, Kyoto, Japan). The HPLC column was not used in this method. The ion abundances were quantified by calculating the signal intensity ratio of the compound to its internal standard.

Analysis was performed by using the analytical conditions described before as follows: mobile phase: acetonitrile:water, 80:20 v/v; flow rate, 0.1 ml/min; injection volume, 10 μl; analysis time, 3 min; ionization mode: ESI (+) and probe voltage, +4.5 kV; nebulizing gas flow, 3.0 L/min; drying gas flow, 20.0 L/min; DL temperature, 300°C; and block heater temperature, 500°C (14). All data were collected and evaluated in terms of multiple reaction monitoring (MRM) for amino acids and acylcarnitines by using the Shimadzu LabSolutions LCMS Version 5.60 SP2 (Shimadzu Corporation, Kyoto, Japan). The Neonatal Solution (Neonatal Mass Screening Software) was used to calculate the concentration of amino acid/acylcarnitines.

### Anthropometric Evaluation

Anthropometric measurements of all newborns were performed by an infantometer (Seca Mod. 207, Germany) (sensitive 0.1 cm). Weight was measured using an electronic scale (Seca GMBH&Co., kg, Hamburg, Germany) (sensitive to 5 g). The SDS of all measurements according to national standards were calculated (13). Ponderal index (PI) of each patient was calculated as [(BW (g)/BL (cm3)] ×100 and grouped as <10th and >10th percentile (15).

### Statistical Analysis

Statistical Package for the Social Sciences software (SPSS version 16.0, Inc. Chicago, Illinois, USA) was used to analyze the data. While evaluating the study values, descriptive statistical methods [mean, standard deviation, median, frequency, percentage, interquartile range (IQR)] were used. The suitability of normal distribution of the quantitative data was tested by the Shapiro–Wilk test and graphical analysis. Student's *t*-test was used for comparisons of normally distributed quantitative variables between two groups, and the Mann–Whitney *U*-test was used for comparisons of not normally distributed quantitative variables between two groups. Pearson chi-square test and the Fisher–Freeman–Halton test were used for comparison of qualitative data. The relationship between data was analyzed using Pearson correlation (correlation coeeficient: *r*). Statistical significance was accepted as *p* < 0.05.

A *post hoc* power analysis was used to determine the statistical power of the study. When the total sample size was taken as 360 for the three independent groups, the power of the study was calculated as 99.2% with an effect size of 0.25 and an alpha level of 5% (G^*^Power 3.1.9.7 for Windows XP).

### Ethics

The study was approved by the Acibadem Mehmet ali Aydinlar University Ethics Committee (ATADEK 2018-19/19). The study was retrospective and did not involve interventions; thus, informed consent from the parents and patients was not obtained. Consent waiver for this study was obtained from the ethics committee.

## Results

Demographic characteristics and birth anthropometry of the study population according to the BW percentile groups as infants with a BW <10 percentiles (SGA, *n* = 69), between 25 and 75 percentiles (AGA, *n* = 168), and >90 percentiles (LGA, *n* = 124) are given in Table 1.

**Table 1:** Demographic characteristics and birth anthropometry of the study population.

	**SGA *n* = 69**	**AGA *n* = 168**	**LGA *n* = 124**	***p*^**1**^**	***p*^**2**^**	***p*^**3**^**
GW (weeks), mean ± SD	39.0 ± 0.9	39.0 ± 0.9	38.6 ± 0.8	0.44	0.09	0.12
Girls, *n* (%)	28 (41%)	63 (38%)	54 (43%)	0.57	0.55	0.42
Vaginal delivery, *n* (%)	17 (25%)	22 (22%)	17 (14%)	0.89	0.06	0.07
Maternal age, mean ± SD	31.4 ± 4.6	31.0 ± 4.6	32.0 ± 4.2	0.67	0.11	0.34
Smoking status, *n* (%)	4 (6%)	19 (11%)	6 (5%)	0.14	0.13	0.37
BW (g), mean ± SD	2,590 ± 230	3,340 ± 180	3,950 ± 240	0.001	0.001	0.001
BW SDS, mean ± SD	−1.75 ± 0.42	0.20 ± 0.25	1.98 ± 0.44	0.001	0.001	0.001
BL (cm), mean ± SD	48.2 ± 1.5	50.9 ± 1.1	52.4 ± 1.36	0.001	0.001	0.001
BL SDS, mean ± SD	−0.81 ± 0.74	0.63 ± 0.52	1.58 ± 0.65	0.001	0.001	0.001
Head C (cm), mean ± SD	32.99 ± 1.26	35.0 ± 1.12	36.0 ± 1.1	0.001	0.001	0.001
Head C SDS, mean ± SD	−1.30 ± 0.92	0.06 ± 0.89	1.21 ± 0.89	0.001	0.001	0.001
Ponderal index, mean ± SD	23.18 ± 2.01	25.34 ± 1.52	27.45 ± 1.83	0.001	0.001	0.001

*GW, gestational week; VD, vaginal delivery; BW, birth weight; SDS, standard deviation score; BL, birth length (crown–heel length); C, circumference; PI, ponderal index; 10p, 10 percentile*.

*p^1^ is between groups 1 and 2; p^2^ is between groups 2 and 3; p^3^ is between groups 1 and 3*.

### Amino Acids

**Figure 1:**
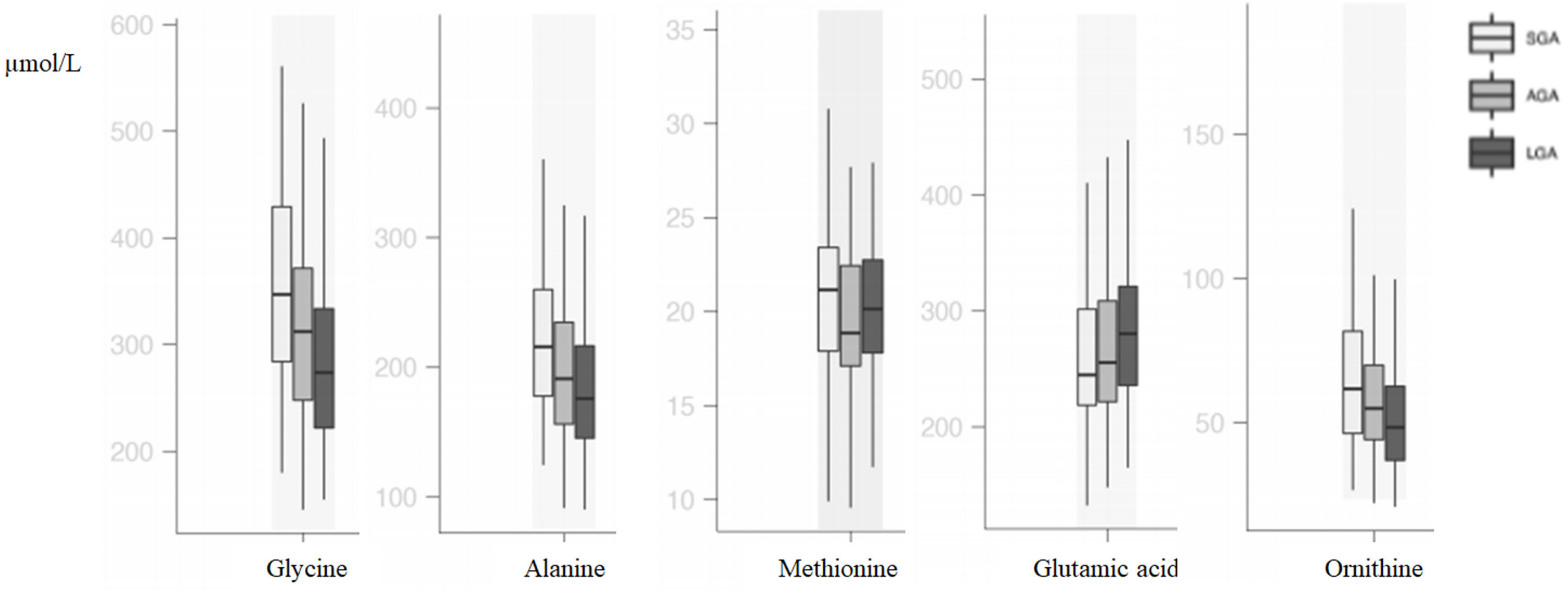
Box and whisker plots of amino acids significantly different among groups.

Compared with AGA newborns, LGA newborns had higher levels of glutamic acid and lower levels of ornithine, alanine, and glycine (*p* < 0.05). SGA newborns had higher alanine and glycine levels when compared with AGA and LGA newborns (*p* < 0.05). Methionine, phenylalanine, and isoleucine levels were higher in SGA when compared with AGA newborns, but no difference was seen between SGA and LGA newborns (*p* < 0.05; Table 2, Figure 1).

**Table 2:** Levels of dried blood spot amino acids.

**Amino acid, units (reference values)**	**SGA *n* = 69**	**AGA *n* = 168**	**LGA *n* = 124**	***p*^**1**^**	***p*^**2**^**	***p*^**3**^**
Alanine, μmol/L (90–900), median (IQR)	215.89 (177.54–261.73)	191.25 (155.84–235.04)	175.93 (145.43–216.83)	**0.006**	**0.01**	**0.001**
Methionine, μmol/L (9.0–65), median (IQR)	21.15 (17.88–23.76)	18.85 (17.10–22.44)	20.14 (17.74–22.78)	**0.04**	0.08	0.41
Phenylalanine, μmol/L (22.9–120), median (IQR)	50.34 (45.21–57.23)	48.57 (42.81–53.82)	48.87 (43.56–56.62)	**0.02**	0.24	0.19
Tyrosine, μmol/L (26.9–275), median (IQR)	76.66 (58.01–104.54)	70.25 (55.42–93.15)	69.25 (53.57–86.47)	0.15	0.65	0.10
Leucine/Isoleucine (IIe), μmol/L (43.4–373), median (IQR)	104.44 (81.74–119.29)	93.66 (83.15–107.72)	95.81 (83.85–109.98)	**0.04**	0.49	0.11
Valine, μmol/L (52.8–250), median (IQR)	92.95 (75.31–106.96)	93.70 (77.67–110.73)	88.22 (76.26–104.21)	0.51	0.22	0.75
Arginine, μmol/L (0.0–50), median (IQR)	8.00 (5.51–11.45)	7.42 (4.91–10.0)	7.46 (4.71–11.02)	0.11	0.82	0.17
Citrulline, μmol/L (2.0–65), median (IQR)	10.54 (8.90–12.33)	10.75 (8.74–12.71)	9.79 (8.11–12.75)	0.93	0.19	0.17
Glycine, μmol/L (105–1,100), median (IQR)	347.09 (284.06–429.99)	312.17 (246.27–371.67)	273.87 (221.45–333.73)	**0.004**	**0.007**	**0.001**
Ornithine, μmol/L (19.7–300), median (IQR)	61.73 (44.62–83.02)	54.93 (44.05–70.40)	48.35 (36.64–63.60)	0.09	**0.005**	**0.001**
Arginino-succinic acid, μmol/L (0.0–0.66), median (IQR)	0.020 (0.010–0.035)	0.027 (0.009–0.052)	0.021 (0.007–0.043)	0.10	0.17	0.79
Glutamic acid, μmol/L (0.0–704), median (IQR)	244.88 (217.50–304.28)	255.49 (220.73–308.93)	280.37 (235.37–321.27)	0.62	**0.04**	**0.04**

*p^1^ is between groups 1 and 2; p^2^ is between groups 2 and 3; p^3^ is between groups 1 and 3*.

*Bold values are emphasizing the statistical significance*.

In the SGA subgroups [< -2 SDS (*n* = 19) vs. −2 SDS-10th (*n* = 50)], infants < -2 SD had significantly higher alanine [263.43 (192.99–291.46) vs. 211.44 (177.05–234.29); *p* = 0.01] and isoleucine levels [110.77 (104.30–141.04) vs. 96.72 (78.06–115.04); *p* = 0.01].

### Acylcarnitine Profiles

**Figure 2:**
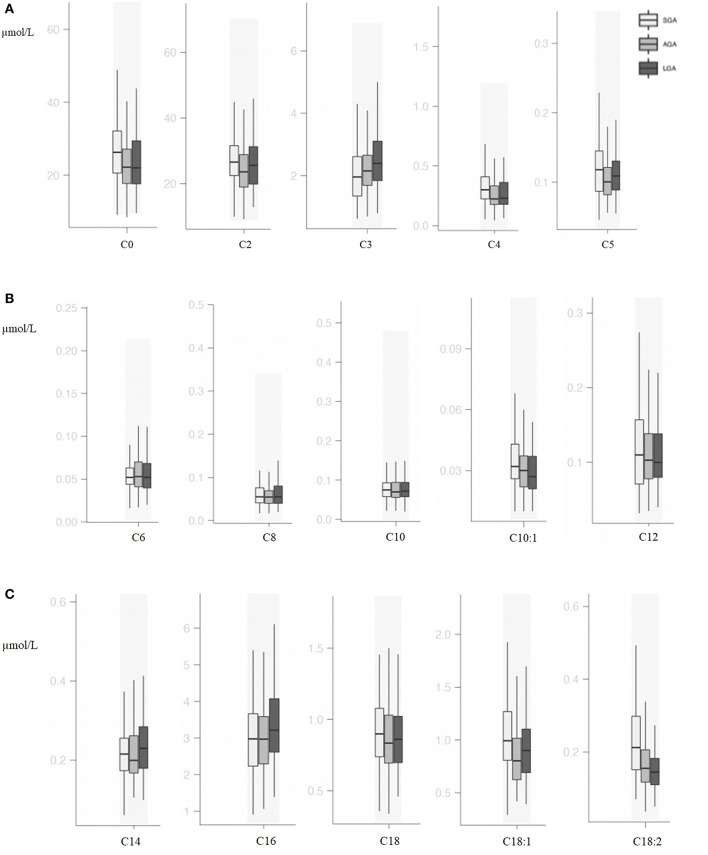
**(A)** Box and whisker plots of free carnitine and short-chain acylcarnitines among groups. **(B)** Box and whisker plots of medium-chain acylcarnitines among groups. **(C)** Box and whisker plots of long-chain acylcarnitines among groups.

**Figure 3:**
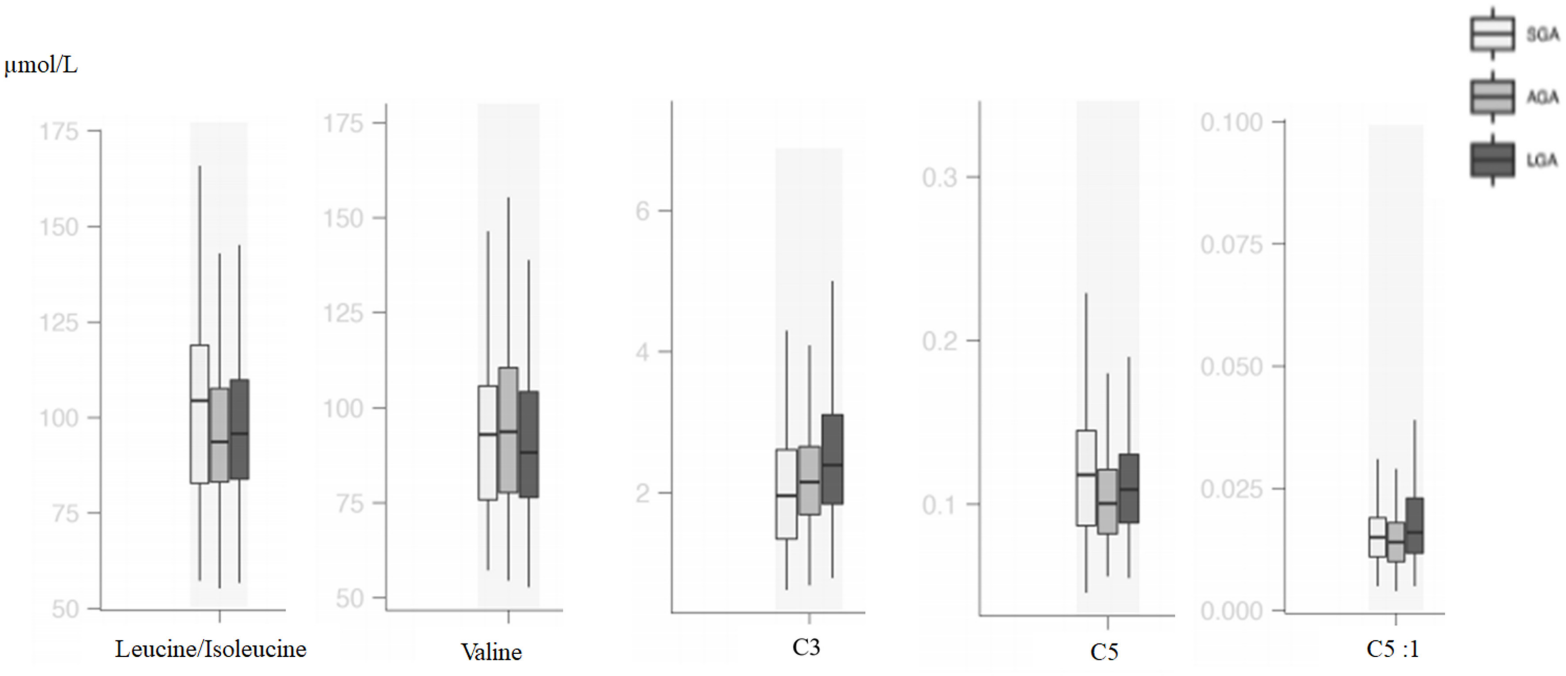
Box and whisker plots of metabolic parameters associated with branched-chain amino acid metabolism among groups.

Compared with AGA newborns, LGA patients had higher levels of C3, C5:1, C16, C14-OH, and C18:1 (*p* < 0.05). On the other hand, LGA patients had lower C16:1-OH levels when compared with AGA patients (*p* < 0.05). Total carnitine, C0, C2, C4, C5, C10:1, C18:1, C18:2, C14-OH, and C18:2-OH levels were significantly higher in SGA infants compared with AGA and LGA infants (*p* < 0.05). C3 and C6-DC levels were lower in SGA patients (*p* < 0.05; Table 3, Figures 2, 3).

**Table 3:** Levels of dried blood spot acylcarnitine.

**Acylcarnitines, units (reference values)**	**SGA *n* = 69**	**AGA *n* = 168**	**LGA *n* = 124**	***p*^**1**^**	***p*^**2**^**	***p*^**3**^**
Free carnitine (C0), μmol/L (8.6–90.0), median (IQR)	26.29 (20.11–32.36)	22.22 (17.69–27.21)	22.03 (17.53–29.43)	**0.002**	0.51	**0.01**
Total carnitine, μmol/L, median (IQR)	60.62 (50.79–77.67)	55.54 (43.71–65.66)	56.98 (47.41–69.41)	**0.003**	0.13	**0.03**
**Short-Chain acylcarnitines**						
Acetyl carnitine (C2), μmol/L (5.0–73.4), median (IQR)	26.55 (22.11–31.81)	23.58 (18.87–28.81)	25.57 (19.81–31.46)	**0.008**	0.06	0.20
Propionyl carnitine (C3), μmol/L (0.0–6.8), median (IQR)	1.96 (1.33–2.62)	2.15 (1.69–2.67)	2.39 (1.83–3.11)	**0.03**	**0.014**	**0.001**
Butyryl carnitine (C4), μmol/L (0.0–1.2), median (IQR)	0.33 (0.21–0.41)	0.22 (0.17–0.33)	0.22 (0.17–0.37)	**0.008**	0.87	**0.01**
Isovaleryl carnitine (C5), μmol/L (0.0–0.6), median (IQR)	0.11 (0.08–0.14)	0.10 (0.08–0.12)	0.10 (0.08–0.13)	**0.004**	0.30	0.29
Tiglyl carnitine (C5:1), μmol/L (0.0–0.13), median (IQR)	0.015 (0.010–0.019)	0.014 (0.010–0.018)	0.016 (0.011–0.023)	0.50	**0.12**	0.17
**Medium-chain acylcarnitines**						
Hexonyl carnitine (C6), μmol/L (0.0–0.21), median (IQR)	0.052 (0.044–0.065)	0.053 (0.041–0.070)	0.052 (0.039–0.068)	0.85	0.58	0.58
Octanoyl carnitine (C8), μmol/L (0.0–0.32), median (IQR)	0.055 (0.040–0.076)	0.054 (0.040–0.069)	0.055 (0.040–0.080)	0.82	0.33	0.59
Decanoyl carnitine (C10), μmol/L (0.0–0.48), median (IQR)	0.075 (0.056–0.093)	0.070 (0.056–0.094)	0.072 (0.057–0.094)	0.79	0.57	0.90
Decenoyl carnitine (C10:1), μmol/L (0.0–0.28), median (IQR)	0.032 (0.025–0.044)	0.030 (0.022–0.037)	0.027 (0.021–0.037)	**0.02**	0.37	**0.004**
Dodecanoyl carnitine (C12), μmol/L (0.0–0.69), median (IQR)	0.11 (0.07–0.15)	0.10 (0.07–0.13)	0.10 (0.08–0.13)	0.65	0.92	0.72
**Long-Chain acylcarnitines**						
Tetradecanoyl carnitine (C14), μmol/L (0.0–0.8), median (IQR)	0.21 (0.16–0.25)	0.19 (0.16–0.26)	0.22 (0.17–0.28)	0.57	0.42	0.14
Tetradecenoyl carnitine (C14:1), μmol/L (0.0–0.6), median (IQR)	0.12 (0.08–0.17)	0.11 (0.09–0.16)	0.13 (0.10–0.18)	0.64	0.17	0.20
Tetradecadienoyl carnitine (C14:2), μmol/L (0.0–0.25), median (IQR)	0.030 (0.023–0.036)	0.027 (0.019–0.036)	0.029 (0.019–0.036)	0.08	0.46	0.33
Palmitoyl carnitine (C16), μmol/L (0.0–8.70), median (IQR)	2.98 (2.23–3.66)	2.97 (2.29–3.58)	3,21 (2.61–4.07)	0.96	0.01	0.06
Palmitoleyl carnitine (C16:1), μmol/L (0.0–1.04), median (IQR)	0.24 (0.18–0.31)	0.23 (0.19–0.29)	0.26 (0.19–0.32)	0.67	0.09	0.35
Stearyl carnitine (C18), μmol/L (0.0–2.24), median (IQR)	0.89 (0.72–1.08)	0.83 (0.69–1.03)	0.86 (0.69–1.02)	0.22	0.67	0.41
Oleyl carnitine (C18:1), μmol/L (0.0–2.8), median (IQR)	0.99 (0.78–1.28)	0.80 (0.62–1.02)	0.90 (0.68–1.11)	**0.001**	0.02	**0.03**
Linolenoyl carnitine (C18:2), μmol/L (0.0–0.9), median (IQR)	0.21 (0.15–0.29)	0.15 (0.11–0.20)	0.14 (0.11–0.18)	**0.001**	**0.08**	**0.001**
**Acylcarnitine esters derived from dicarboxylic acids-DC**						
Glutaryl carnitine (C5-DC), μmol/L (0.0–0.21), median (IQR)	0.072 (0.051–0.092)	0.074 (0.058–0.096)	0.080 (0.061–0.107)	0.58	0.67	0.04
Methylglutaryl carnitine (C6-DC), μmol/L (0.0–0.20), median (IQR)	0.026 (0.020–0.037)	0.030 (0.025–0.040)	0.033 (0.026–0.042)	**0.01**	0.11	**0.02**
**Acylcarnitine esters derived from hydroxylated acids-OH**						
3-OH butyryl carnitine (C4-OH), μmol/L (0.0–0.48), median (IQR)	0.15 (0.11–0.20)	0.14 (0.10–0.19)	0.16 (0.12–0.23)	0.45	0.01	0.23
3-OH isovaleryl carnitine (C5-OH), μmol/L (0.0–0.80), median (IQR)	0.11 (0.09–0.13)	0.11 (0.08–0.12)	0.11 (0.08–0.14)	0.55	0.36	0.88
3-OH tetradecanoyl carnitine (C14-OH), μmol/L (0–0.12), median (IQR)	0.029 (0.023–0.034)	0.026 (0.020–0.031)	0.026 (0.020–0.032)	**0.04**	0.86	0.04
3-OH palmitoyl carnitine (C16-OH), μmol/L (0.0–0.1), median (IQR)	0.036 (0.027–0.044)	0.036 (0.025–0.041)	0.034 (0.027–0.042)	0.53	0.57	0.91
3-OH palmitoleyl carnitine (C16:1-OH), μmol/L (0.0–0.18), median (IQR)	0.030 (0.022–0.039)	0.028 (0.021–0.034)	0.025 (0.019–0.031)	0.09	**0.05**	**0.002**
3-OH oleyl carnitine (C18:1-OH), μmol/L (0.0–0.1), median (IQR)	0.015 (0.012–0.020)	0.017 (0.012–0.019)	0.017 (0.012–0.022)	0.85	0.17	0.29
3-OH linonenoyl carnitine (C18:2-OH), μmol/L (0.0–0.1), median (IQR)	0.013 (0.010–0.017)	0.012 (0.010–0.016)	0.012 (0.010–0.016)	**0.03**	0.08	0.13

*p^1^ is between groups 1 and 2; p^2^ is between groups 2 and 3; p^3^ is between groups 1 and 3.*

*Bold values are emphasizing the statistical significance*.

In the SGA subgroups (< -2 SDS vs. −2 SDS-10th), infants < -2 SD had significantly higher C0 [31.88 (20.59–48.87) vs. 23.57 (19.33–31.10); *p* = 0.03] and C16:1OH [0.02 (0.01–0.04) vs. 0.02 (0.02–0.03); *p* = 0.02], C18:2 [0.31 (0.21–0.41) vs. 0.17 (0.14–0.23); *p* = 0.001] and lower C4 levels [0.28 (0.19–0.31) vs. 0.32 (0.23–0.46); *p* = 0.02].

### Correlations Between Amino Acids and Carnitine/Acylcarnitines

**Figure 4:**
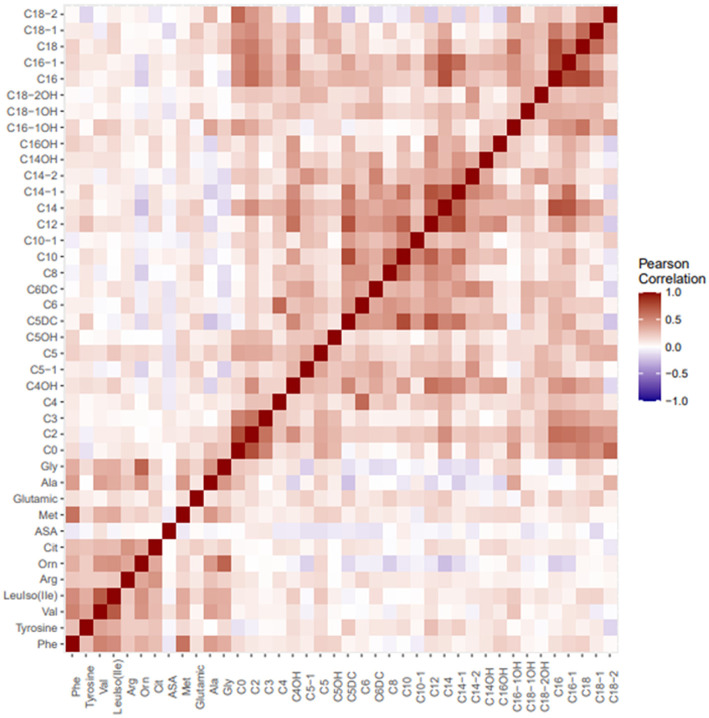
Heatmap visualizing correlations between metabolites in tandem MS/MS amino acid and carnitine profile.

In all the study population, there were positive correlations between free carnitine and phenylalanine (*r* = 0.149, *p* = 0.005), arginine (*r* = 0.126, *p* = 0.017), methionine (*r* = 0.146, *p* = 0.006), alanine (*r* = 0.282, *p* = 0.001), and glycine (*r* = 0.233, *p* = 0.001). Correlations were also shown as heatmap in Figure 4.

### Correlations Between PI and Amino Acids and Carnitine/Acylcarnitines

In all the study population, there were positive correlations between PI and C3 (*r* = 0.155, *p* = 0.003), C5-DC (*r* = 0.116, *p* = 0.029), C14 (*r* = 0.106, *p* = 0.045), and C14:1 (*r* = 0.112, *p* = 0.033); on the other hand, a negative correlation between PI and ornithine (*r* = −0.184, *p* = 0.001), alanine (*r* = −0.226, *p* = 0.001), glycine (*r* = −0.199, *p* = 0.001), C16:1-OH (*r* = −0.163, *p* = 0.002), and C18:2 (*r* = −0.178, *p* = 0.001) was seen.

## Discussion

Being born SGA or LGA has been linked to a number of long-term risks for developing chronic metabolic diseases. The early origin of these diseases explained by the Barker hypothesis points to the intrauterine environment as the main determinant of these conditions (16). The metabolic profile of newborn infants can be an indicator of intrauterine problems originating from the mother, offspring, or both. Metabolomics under the influence of intrauterine life can shed light on the metabolic status of later life.

In our study, alanine, glycine, methionine, phenylalanine, ornithine, and isoleucine were higher in SGA infants and glutamic acid was higher in LGA infants. Vidarsdottir et al. (6) have analyzed 6,131 babies, of which 36 of the babies were LBW (below 2,500 g) and 37 of them were extremely macrosomic. They reported that alanine and threonine were higher in LBW babies and glutamic acid was higher in extremely macrosomic babies similar to our findings. In that study, babies were only classified due to their BW without reporting their gestational age status. Liu et al. (17) evaluated 60 SGA and 60 AGA babies and found lower levels of alanine, homocysteine, methionine, ornithine, serine, and tyrosine in babies below the 3rd percentile. Although they did not give the comparison of SGA (defined as <10th percentile) and AGA babies, according to their results, alanine and ornithine seem to be higher in SGA babies. Manta-Vogli et al. (18) enrolled 2,000 neonates and found that ornithine, glutamine, and glutamic acid levels were inversely related with BW and concluded that this difference was due to breastfeeding, intestinal maturation, and muscle mass of the babies. In another study, SGA neonates were found to have higher alanine, tyrosine, citrulline, glycine, ornithine, and proline levels (19). In a recent animal study, increased alanine production and decreased skeletal muscle amino acid uptake were shown in growth-restricted sheep fetuses (20). In the early days of life, energy metabolism is in a catabolic state, and after a process of adaptation to extrauterine life with feeding, it reaches the anabolic state. In this catabolic state, proteins are broken down and released glucogenic amino acids are used to generate energy. Glutamic acid works in energy metabolism linking carbohydrate and amino acid metabolism via oxidative pathways (21). Alanine is the major predictor of lactate metabolism and facilitates glucose metabolism, and higher levels of alanine have been reported to increase the risk of diseases related to metabolic syndrome in later life (22). The consequences of these metabolic processes are thought to be the cause of amino acid differences identified in our study. It can be speculated that amino acids are consumed to generate energy rather than protein accretion in SGA babies.

Metabolites that originated from amino acid catabolism take place in the acylcarnitine profile, especially as odd-numbered chain acylcarnitines (C3, C5, C5-OH, C5-DC). C3 and C5 are derived from leucine, isoleucine, and valine catabolism, and these are all branched-chain amino acids (BCAAs) (23). In several studies, BCAAs C3 and C5 were usually mentioned as biomarkers for insulin resistance (24). In a large cohort, it was shown that maternal BMI was associated with cord blood BCAAs and their catabolites C3 and C5 (25). Analysis of neonatal plasma at 48 h of life was found to show higher C5 relative to neonatal adiposity in offsprings of overweight/obese, but not normal-weight, mothers (26). C5 is a marker of insulin resistance and related with compensatory increased BCAA catabolism (27, 28). In our study, LGA patients had higher C3 and C5:1 levels, and SGA patients had higher C5 and isoleucine levels; similar to previous studies, differences in BCAA metabolism were also shown.

Carnitine and acylcarnitines are critical substances for cellular energy metabolism. The expression of a carnitine transporter gene, *SLC22A5*, in human placenta suggests that the main source of carnitine in fetal life is the mother *via* transplacental transport (29). Although this is supported by several studies, it has been reported that a fetus has γ-butyrobetaine dioxygenase (BBD) activity and human fetal placental unit may have a role in carnitine biosynthesis especially when maternal carnitine supply is not sufficient (30). The even-numbered chain acylcarnitines, especially from C6 to C20, are dominantly the products of fatty acid β-oxidation. In addition to β-oxidation, C2 and C4 are produced mostly by carbohydrate catabolism and amino acid metabolism, respectively. Lipotoxicity and impairments of β-oxidation of fatty acid have an important role in the development of insulin resistance. Therefore, acylcarnitine profile may reflect insulin action (23, 31). Our results showed that SGA babies have significant differences in carnitine and acylcarnitine profiles. SGA newborns have higher levels of C0 and total carnitine, short-chain acylcarnitines (C2, C4), medium-chain acylcarnitines (C10:1, C14-OH), and long-chain acylcarnitines (C18:1, C18:2, C18:2-OH). Similar to our findings, Liu et al. (19) found increased levels of other acylcarnitines in SGA patients. In a recent study, C0 was found to be higher in babies with birth weight between 2,000 and 2,500 g; however, the authors did not mention the gestational ages and the SGA or LGA status of these babies (7). Sanches-Pintos et al. (32) demonstrated higher carnitine levels in LGA newborns than in AGA, and the subgroup analysis of LGA newborns showed that there was no difference in babies with mothers having GDM. However, SGA newborns have also higher levels of total carnitines and short-chain acylcarnitines, and an increased pattern of acylcarnitines was more evident in the SGA group. Fatty acid oxidation rate is low in the fetus (33) and becomes important due to the cessation of transplacental glucose transfer immediately after birth and the insufficiency of glycogen stores in the newborn infant. It may be thought that high free carnitine levels may be a associated with increased fatty acid oxidation in SGA infants. On the other hand, impaired fatty acid metabolism might cause an increase in short-chain acylcarnitines and other acylcarnitines in SGA babies. Increased short-chain fatty acids have also been demonstrated in adult diabetics or prediabetic individuals (34). In a study involving the analysis of plasma at 48 h of life, markers of incomplete mitochondrial lipid oxidation (medium-chain and dicarboxylic acylcarnitines) relative to neonatal adiposity in the offspring of overweight/obese, but not normal-weight, mothers were found (26). Amino acids and acylcarnitines have been studied in various populations including children and adults with obesity and type 2 DM, and associations have been reported (24, 35). They have also been shown to be important in the metabolic status of the newborn (6). Considering the risks of developing DM and metabolic syndrome in SGA babies later in life, it can be thought that these metabolic markers start to increase from birth.

The limitations of this study include the retrospective nature of the study and the relatively low number of subjects. The second limitation is that we do not have Doppler sonographic evaluations of fetal growth to define intrauterine growth restriction. However, our results will be a pioneer for further prospective studies in which intrauterine growth-restricted newborns are evaluated.

In this study, differences in metabolomics reflecting the energy metabolism in infants with intrauterine growth problems in early postnatal period have been shown. These differences might be the footprints of metabolic disturbances later in life. Further prospective studies including long-term follow-up from gestation to adulthood are needed.

## Data Availability Statement

The datasets presented in this study can be found in online repositories. The name of the repository and accession number can be found at: MetaboLights, https://www.ebi.ac.uk/metabolights/search, MTBLS2376.

## Ethics Statement

The study was approved by the Acibadem Mehmet ali Aydinlar University Ethics Committee (ATADEK 2018-19/19). Written informed consent from the participants' legal guardian/next of kin was not required to participate in this study in accordance with the national legislation and the institutional requirements.

## Author Contributions

SB, NY, EA, MK, FB, AK, and MS: medical practices. SB, SA, and ME: concept. SB, SA, NY, and ME: design. SB, BG, TD, EA, MH, EB, DK, MK, ZA, and GE: data collection or processing. SB, SA, and TD: analysis or interpretation. SB, SA, NY, ME, FB, AK, and MS: literature search. SB, SA, NY, FB, AK, and MS: writing. All authors discussed the results, commented on the manuscript, accepted responsibility for this submitted manuscript, and approved for its submission.

## Conflict of Interest

The authors declare that the research was conducted in the absence of any commercial or financial relationships that could be construed as a potential conflict of interest.
